# Assessing Coverage of Population-Based and Targeted Fortification Programs with the Use of the Fortification Assessment Coverage Toolkit (FACT): Background, Toolkit Development, and Supplement Overview^1^,^2^,^3^

**DOI:** 10.3945/jn.116.242842

**Published:** 2017-04-12

**Authors:** Valerie M Friesen, Grant J Aaron, Mark Myatt, Lynnette M Neufeld

**Affiliations:** 4 Global Alliance for Improved Nutrition, Geneva, Switzerland; and; 5 Brixton Health, Llawryglyn, United Kingdom

**Keywords:** large-scale food fortification, staple foods, infant and young child nutrition, program coverage, toolkit

## Abstract

Food fortification is a widely used approach to increase micronutrient intake in the diet. High coverage is essential for achieving impact. Data on coverage is limited in many countries, and tools to assess coverage of fortification programs have not been standardized. In 2013, the Global Alliance for Improved Nutrition developed the Fortification Assessment Coverage Toolkit (FACT) to carry out coverage assessments in both population-based (i.e., staple foods and/or condiments) and targeted (e.g., infant and young child) fortification programs. The toolkit was designed to generate evidence on program coverage and the use of fortified foods to provide timely and programmatically relevant information for decision making. This supplement presents results from FACT surveys that assessed the coverage of population-based and targeted food fortification programs across 14 countries. It then discusses the policy and program implications of the findings for the potential for impact and program improvement.

## Introduction

Fortification is a widely used intervention strategy to increase micronutrient intake in the diet. Fortification strategies can be population based or targeted. Population-based fortification strategies are designed to reach the general population through food vehicles that are regularly and frequently consumed by large segments of the population (i.e., staple foods and/or condiments). The implicit assumption is that those at risk of inadequate micronutrient intake will be reached while avoiding toxicity in those with an adequate intake and/or micronutrient status. Targeted fortification strategies are designed to reach a particular population group with the use of specific interventions with products that are fortified at amounts required to meet dietary gaps (e.g., complementary foods for infants and young children, foods designed for pregnant and/or lactating women, emergency rations, or point-of-use fortification such as micronutrient powders in which nutrients are added immediately before consumption).

Fortification, whether population based or targeted, is conceptually simple. Several conditions must be in place, however, for programs to be impactful. Much of this has been outlined in global recommendations (1) and good practice guidance (2). Briefly, considering a typical program cycle at the design phase, the intervention should be justified by demonstrated micronutrient needs in the target population and an assessment of vehicle suitability. At the implementation phase, the intervention should be well designed, and ongoing program monitoring is essential to identify and implement timely course correction, improve the quality of implementation, and measure progress against program goals. At the evaluation phase, the impact on biological or functional outcomes should be considered only if data collected during the implementation phase suggest high enough coverage and utilization for such an impact to be plausible. Despite the importance of these conditions, gaps in the design, implementation, and evaluation of fortification programs are common (3), and information on coverage and utilization is rarely available (4, 5). In particular, many fortification programs have forgone household-level coverage assessments (4). Reasons for this include the lack of standardized, fit-for-purpose tools to facilitate the collection of quality and timely information on coverage and utilization at the population level and to provide a potential for comparisons across multiple settings.

## Tools and Methods to Inform Fortification Program Design and Assess Program Performance

Some tools are available to guide fortification programmers, but their utility to assess program coverage is limited. For population-based staple food fortification approaches, the Fortification Rapid Assessment Tool was developed in the late 1990s to simplify the collection of information required to select appropriate food vehicles and set fortification levels (6) with the use of modified 24-h recall and FFQ methods. Several countries, particularly in Africa, have used Fortification Rapid Assessment Tool surveys to plan for national fortification programs (7). This method was adapted and used for assessing program coverage in at least one country (8). For some food vehicles, including salt, oil, and wheat flour, detailed monitoring manuals have been developed to encourage standardized and appropriate regulatory monitoring practices (9–11). Regulatory monitoring focuses on the compliance of industry with fortification standards and laws, and, as such, does not include specifics related to coverage and utilization assessment. The Fortification Monitoring and Surveillance tool was designed to track trends in the effectiveness of flour fortification programs over time, relying mainly on data generated from routine program monitoring, as well as tracking of hemoglobin concentration from surveillance systems (12). Tools to assist program managers working with targeted fortification interventions are more limited. For home fortification interventions, the CDC and the Home Fortification Technical Advisory Group recently developed a monitoring manual that provides technical guidance on how to develop and implement monitoring systems to track home fortification programs (13). Similar to the tools described for population-based fortification programs, little information is provided related to methodologies for assessment of coverage and utilization.

The Global Alliance for Improved Nutrition (GAIN) has supported a large portfolio of population-based and targeted fortification interventions since being founded in 2002 (14). In an effort to prioritize and standardize coverage assessments, GAIN developed a Fortification Assessment Coverage Toolkit (FACT) to carry out coverage assessments in both population-based (i.e., staple foods and/or condiments) and targeted (e.g., infant and young child) fortification programs. The toolkit was designed to facilitate coverage and utilization assessments of programs, thereby filling in important gaps in the availability of standardized and program-oriented tools for fortification stakeholders. The ultimate goal of this body of work is to set a precedent for prioritizing coverage assessments of fortification programs that provide timely and relevant information for decision making related to program improvement.

The FACT methods focus on 3 key areas: *1*) identifying and classifying at-risk population subgroups with the use of diverse measures of vulnerability that are associated with poor nutrition and health outcomes in low-resource settings (e.g., poverty, rural residence, poor dietary diversity, and poor infant and young child feeding practices); *2*) assessing coverage and utilization of fortified food vehicles (e.g., staple foods in large-scale fortification programs or fortified foods targeted to specific population groups); and *3*) assessing the quality of fortified foods to determine the adequacy of fortification levels at the local market and/or the household level independently of routine monitoring activities. All survey modules (i.e., question and indicator sets) were taken or adapted from validated instruments where available (15–17). The initial draft of the FACT toolkit detailing design elements and research approach was prepared in May 2013 as part of a grant deliverable to the Bill & Melinda Gates Foundation. The toolkit was reviewed by independent subject-matter experts commissioned by the Bill & Melinda Gates Foundation and then further refined based on feedback.

Timeliness of results often poses a challenge to program managers to use research for decision making (18). Considerations were therefore made to ensure that the toolkit could be implemented, analyzed, and reported rapidly while maintaining rigor and low cost. A pilot survey was conducted in 3 districts in eastern Ghana in July 2013, taking advantage of an already planned coverage assessment of a targeted fortification program for infants and children (15). The instrument was finalized during a 3-d technical workshop in September 2013.

## Overview of Supplement

The purpose of this supplement is to bring together information generated from FACT surveys to date. The articles in this supplement demonstrate the applications across different countries and contexts, and provide insights into how this information has been and can be used to improve program decision making. Individual surveys were designed and implemented in partnership with reputed in-country and international technical partners. In all cases, the results were shared in-country with government, industry, and other partners, and have been used to identify and address implementation challenges. Detailed country-specific papers have been published elsewhere (15, 16, 19–22) or are in preparation.

The first paper in the supplement, *Coverage of Large-Scale Food Fortification of Edible Oil, Wheat Flour, and Maize Flour Varies Greatly by Vehicle and Country but Is Consistently Lower among the Most Vulnerable: Results from Coverage Surveys in 8 countries*, presents population-based food fortification program coverage results from 8 FACT surveys conducted from 2013–2015 (17). Results focus on household coverage of edible oil and wheat and maize flours. Data are from Bangladesh, Côte d’Ivoire (Abidjan), India (Rajasthan), Nigeria (Kano and Lagos), Senegal, South Africa (Gauteng and Eastern Cape), Tanzania, and Uganda. The article presents implications in these countries to improve program decision making and summarizes lessons learned and potential areas for further development of the FACT in its application to population-based food fortification programs.

The second paper, *Coverage of Nutrition Interventions Intended for Infants and Young Children Varies Greatly Across Programs: Results from Coverage Surveys in 5 Countries,* presents results on individual coverage of targeted fortification programs from 11 surveys conducted across 5 countries from 2013 to 2015 (23). Results focus on coverage of fortified complementary foods and food supplements as part of fortification interventions for infants and young children. Data are from Bangladesh, Côte d’Ivoire, Ghana, India (Telangana), and Vietnam. The article reviews the implications of the specific programs’ findings and for further application of the FACT to fortification programs targeted at infants and young children.

The third paper, *Household Coverage with Adequately Iodized Salt Varies Greatly between Countries and by Residence Type and Socioeconomic Status within Countries: Results from 10 National Coverage Surveys,* presents program coverage results from 10 countries with mandatory universal salt iodization programs (24). Results focus on household coverage of iodized and adequately iodized salt by country, including an investigation of the relation between coverage and socioeconomic status and residence type (i.e., urban compared with rural). These surveys were implemented in 8 of the Universal Salt Iodization GAIN-UNICEF Partnership Project countries (Bangladesh, Ethiopia, Ghana, India, Indonesia, Niger, the Philippines, and Senegal), in addition to 2 national FACT surveys in Tanzania and Uganda.

In the fourth and final paper, *Coverage and Utilization in Food Fortification Programs: Critical and Neglected Areas of Evaluation*, the authors highlight key messages from the preceding papers and discuss in-depth the policy and program implications of the body of work (25). The paper also provides reflections on the strengths and potential areas for improvement of the FACT and its potential application in a more comprehensive system to track coverage and utilization of nutrition interventions.
